# Clotting Properties of *Onopordum tauricum* (Willd.) Aqueous Extract in Milk of Different Species

**DOI:** 10.3390/foods9060692

**Published:** 2020-05-27

**Authors:** Massimo Mozzon, Roberta Foligni, Cinzia Mannozzi, Federica Zamporlini, Nadia Raffaelli, Lucia Aquilanti

**Affiliations:** Department of Agricultural, Food and Environmental Sciences, Università Politecnica delle Marche, Via Brecce Bianche 10, 60131 Ancona, Italy; m.mozzon@staff.univpm.it (M.M.); r.foligni@staff.univpm.it (R.F.); f.zamporlini@staff.univpm.it (F.Z.); n.raffaelli@staff.univpm.it (N.R.); l.aquilanti@staff.univpm.it (L.A.)

**Keywords:** rennet, *Onopordon tauricum*, thistle crude extract, milk clotting activity, response surface methodology, goat’s milk, ewe’s milk, vegetable coagulant, plant proteases

## Abstract

Plant proteases used in cheesemaking are easily available and could increase the acceptability of cheeses, otherwise hindered by ethical issues (e.g., religions, dietary habits, aversion to genetically engineered food and food ingredients). The milk clotting potential of *Onopordum tauricum* (Willd.) aqueous extract as an alternative to animal rennet was assessed for the first time in milk of different species (ewe, goat, cow). Among the aerial anatomical parts, i.e., receptacle, leaves, stems, and flowers, only the latter ones showed clotting properties. A response surface methodology (RSM) was used to explore the effects of three independent variables (temperature, pH, volume of coagulant) on the milk clotting activity (MCA) of the flower extract. A second-order polynomial model adequately described the experimental data and predicted a temperature value of 55 °C, a pH value of 4.9–5.7, and a volume of coagulant of 300–500 μL (added to 5 mL of milk) as optimal conditions to maximize the MCA. At a 35 °C temperature and natural milk pH of 6.7–6.8, the estimated MCA of the *O. tauricum* extract was 72–87, 69–86, and 75–151, in goat’s, ewe’s, and cow’s milk, respectively. In comparison, the MCA of calf rennet was 5.4–4.9, 3.3–14.7, and 4.9–16.7 times higher than that of the plant extract in goat’s, ewe’s, and cow’s milk, respectively.

## 1. Introduction

In Southern European and Western African countries, plant proteases have been used for centuries as milk coagulants in cheesemaking, especially for the curdling of raw ovine and caprine milks. Proteolytic activities have been found in the leaves, fruits, flowers, stems, seeds, and latex of several herbs, woody plants, and trees [1]. Even if the use of vegetable rennet is geographically circumscribed and limited to few traditional products, the scarcity and high price of conventional animal rennet have led to a growing interest in the vegetable sources of milk-clotting enzymes. Several factors have been brought up to explain the reduced supply and demand for traditional animal rennet, i.e., ethical issues (religions, dietary habits), ban of recombinant chymosin in some countries, increase in cheese production worldwide, and incidence of bovine spongiform encephalopathy [2].

In western and central Mediterranean areas, crude aqueous extracts with milk clotting properties are traditionally prepared from spontaneous herbaceous plants commonly referred to as “thistles” and scientifically ascribed to different genera within the family *Asteraceae*, namely *Cynara*, *Silybum*, *Centaurea*, *Carlina*, *Cirsium*, and *Onopordum* [1,2,3]. Spain and Portugal have the largest variety and production of raw ewe’s and goat’s milk cheeses using vegetable coagulants, which are mainly produced on an artisanal scale (Serra de Estrela, Serpa, Azeitão, Nisa, Castelo Branco, Évora, Casar de Cáceres, Torta del Casar, Los Pedroches, La Serena, Los Ibores, Flor de Guía) [2]. Some of them have been granted a Protected Designation of Origin (PDO) in the European Union [4]. Seasonal climatic variations can strongly influence the yield of the plant biomass, thus making the manufacture of these cheeses an occasional and unpredictable event [5].

Studies performed so far have shown that thistle extracts contain several proteases with different enzymatic properties, distinct substrate specificities, and structural properties. Most of the enzymes so far used as milk coagulants are aspartic proteases, which are more active at acidic pH values and are specifically inhibited by pepstatin, but other enzymes such as cysteine and serine proteases have also been reported to possess the ability of clotting milk under proper conditions [1].

*Cynara cardunculus* is by far the most studied species and the most exploited thistle in cheesemaking [2,6,7], but chemical, technological, and nutritional traits of other thistle species have not been fully described yet. Particularly, *Onopordum* spp. have been scarcely studied for their coagulant properties. A partially purified enzyme preparation (“onopordosin”) was obtained from *O. acanthium* L. (cotton thistle, Scotch thistle) flowers. The main active component in the extract from the latter thistle was an aspartic protease, which is characterized by an isoelectric point of 4.4 [8]. The seeds, flowers, and leaves of *O. turcicum* Danin were also found to contain proteolytic enzymes able to coagulate milk [9,10], but *Onopordum tauricum* Willd. (Taurian thistle, bull cottonthistle) is still unexplored from this viewpoint. This is a biennial thistle with a spiny stem up to 2 m tall, bringing spiny, triangular-lobed leaves. The inflorescence (hemispherical flower head, 4–6 cm in diameter) is made up of pink-purple tubular flowers up to 3 cm long. A medicinal use of *O. tauricum* was first recorded in Turkey: a decoction of fruits has been reported to stimulate the flow of bile from the liver and to treat diabetes [11,12]. However, very few data are available about the chemical and nutraceutical traits of Taurian thistle. Phytochemical studies revealed the presence of prebiotics (fructans, inulin) and antioxidants (polyphenols) in ethanol and water extracts obtained from flower heads [13]. Bruno et al. [14] reported the presence of ten sesquiterpene lactones, including a new elamanolide and four new eudesmanolides, flavonoids (apigenin, acacetin, luteolin, hispidulin, nepetin, apigenin 7-O-glucoside, luteolin-7-glucoside), and derivatives of cinnamic acid (caffeic acid, chlorogenic acid) in the chloroform extract of the leaves of *O. tauricum*. Targan et al. [15] analyzed the macro (Na, Mg, and Ca) and trace elements (Li, Fe, Zn, Mn, Se, Al, V, Cr, Ni, Cu, Pb, As, Co, Cd, and Hg) by inductively coupled plasma-mass spectrometry (ICP-MS), after microwave digestion of the aerial parts. Lastly, gas chromatographic analysis showed that linoleic acid is the most abundant component of *O. tauricum* seed oil [16].

No literature data are currently available about the proteases of Taurian thistle and their behavior as milk clotting agents. In this context, the highly specific caseinolytic activity of the aqueous extract from flowers of spontaneously grown *O. tauricum* was characterized for the very first time in milk of different species (ewe, goat, cow). A response surface methodology (RSM) approach was used to study the effect of the curdling variables (temperature, pH, amount of enzymatic extract) on the technological performance of the thistle extract. A comparison with the performance of commercially available calf rennet was also carried out.

## 2. Materials and Methods

### 2.1. Plant Material and Crude Extract Preparation

**Figure 1:**
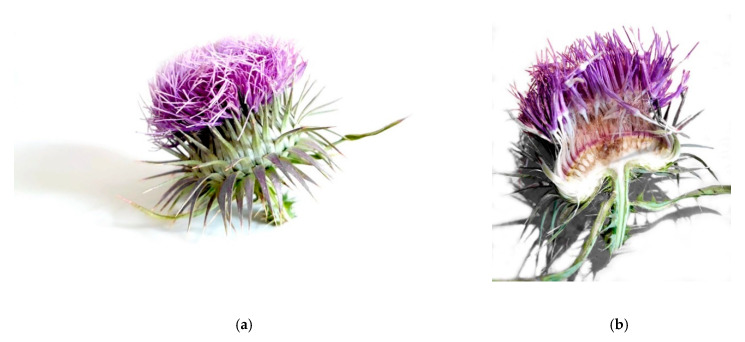
Inflorescence (flower head) of *Onopordum tauricum* (**a**) whole; (**b**) section.

Spontaneously grown *O. tauricum* plants were collected in July 2019 along the outer fringes of the Monti Sibillini National Park, which extends in the hearth of Italy, between the Marche and Umbria regions. Tubular flowers (Figure 1) were manually separated from receptacle immediately after harvesting, and macerated in demineralized water (1:10 w/v) for 24 h at 4 °C. The liquid phase was recovered by filtration through a muslin cloth and subsequent centrifugation (5000× *g*, 10 min). Finally, the aqueous crude extract (CE) was freeze-dried (VirTis Advantage benchtop freeze dryer, Steroglass S.r.l., Perugia, Italy) and stored at −20 °C until it was used. At the time of use, the dried extract was reconstituted in demineralized water 1:10 w/v (reconstituted extract, RE).

The milk clotting performance of RE was compared with a commercial liquid preparation of calf rennet (CR) (51 International Milk Clotting Units, IMCU) provided by Caglificio Clerici (Como, Italy).

### 2.2. Substrates Preparation and Chemical Characterization

Partially skimmed UHT milks from cow and goat were purchased in a local grocery store. Crude ewe’s milk was collected in a local farm (Azienda Agricola Zerbino Francesco, Senigallia, Italy), immediately refrigerated at 4 °C, and transferred to the laboratories of Università Politecnica delle Marche, where it was skimmed by centrifugation at 5000× *g* and 30 °C for 10 min. All skimmed milks were freeze-dried, and milk powders were stored under vacuum at −20 °C. Dry matter percentages were calculated on the basis of the amount of the freeze-dried products. Milk powders were analyzed for fat, protein, and ash contents, according to the procedures described in Roncolini et al. [17], while lactose content was calculated by subtracting the quantified milk nutrients from the total solid content. 

Milk powders were reconstituted in different buffer solutions (see Section 2.3.3.), based on their dry matter contents. To prepare the buffers, a solution of sodium acetate of 100 mM was adjusted to the desired pH values (4.5, 5.0, 5.5, 6.0, 6.5) by adding concentrated acetic acid. The pH values of reconstituted milks were checked by a benchtop pH meter equipped with a glass electrode (Hanna Instruments, Padova, Italy) and the actual pH values were used for modeling the caseinolytic activity of *O. tauricum* proteases.

### 2.3. Characterization of Thistle Extracts

#### 2.3.1. Total Protein Content

The total protein amount of RE and CR was determined according to the Coomassie blue dye binding method [18], using the Bio-Rad (Bio-Rad Laboratories S.r.l, Milan, Italy) ready-to-use reagent. A set of bovine serum albumin (Merck KGaA, Darmstadt, Germany) solutions (0.2–0.9 mg/mL) was used for calibration. Absorbance readings at 595 nm were carried out by using a UV-1800 Shimadzu (Kyoto, Japan) spectrophotometer.

#### 2.3.2. Total Polyphenol Content

The dried plant extract (100 mg) was dissolved in a methanol/water mixture 80:20 v/v (5 mL). The total polyphenol content was determined according to the Folin–Ciocalteu method, as described in Savini et al. [19]. Gallic acid (Sigma-Aldrich, Milan, Italy) was used as an external standard.

#### 2.3.3. Milk Clotting Activity (MCA) Assay

A response surface methodology (RSM) [20] was used to explore the effects of three independent variables (temperature, pH, volume of RE) on the measured parameter (MCA) and the relationships among the explanatory variables. The software JMP Version 11.0.0 (SAS Institute Inc., Cary, NC, USA) was used to both design the experimental plan and analyze the data matrix. Temperature and volume of extract were selected as continuous factors in the range 35–55 °C and 300–500 µL, respectively, while pH was selected as 5-level discrete factor (4.5, 5.0, 5.5, 6.0, 6.5). The selection of ranges of each factor was based on preliminary experimental results. The D-optimal criterion was used for designing the experiment, to obtain the maximum amount of useful information in a reasonable number of experiments to run, including two replicates of each run. The software generated 20 experiments that were carried out in double (Table S1). A second-order response surface, according to the following equation, was used to fit the experimental data matrix:

(1) $${Y=b}_{0}+\sum_{i=1}^{3}b_{i}X_{i}+\sum_{i=1}^{3}b_{\mathrm{ii}}X_{i}^{2}+\sum_{i\neq j=1}^{3}b_{\mathrm{ij}}X_{i}X_{j}$$

where Y is the response variable (MCA); X_i_, X_j_ are the coded values of the input variables (temperature, volume of coagulant, pH); and b_0_, b_i_, b_ii_, and b_ij_ are the regression coefficients for the intercept, linear, quadratic, and interaction terms, respectively.

The experiments were randomized to minimize the effects of unexplained variability. The experimental plan was also carried out using a commercial liquid preparation of CR. The volumes of CR used in each run were adjusted to have the same amounts of total proteins that were in the used volumes of RE.

For the clotting activity determination, 5 mL of reconstituted milk was transferred into a clean and dry test tube. A calcium chloride (Sigma-Aldrich, Milan, Italy) solution (500 g/L) was added to the substrate to achieve the final concentration of 10 mM. The assay tube was allowed to equilibrate for 5 min at the desired temperature in an M20 thermostatic water bath (Lauda-Königshofen, Germany) before the addition of the RE. The time of first visual appearance of flocks on the wall of the test tube was recorded for each run. A Soxhlet’s unit-related definition was used for quantifying the clotting activity. One unit of milk-clotting activity was arbitrarily defined as the volume of milk that can be clotted by one volume unit of RE in 40 min in the assay conditions of pH and temperature [21]:

(2) $$\mathrm{MCA}\;\left(U\right)=\frac{2400}{T}\cdot \frac{S}{E}$$

where T is the clotting time (s); E is volume of RE (mL); and S = volume of milk (mL).

To confirm the enzymatic milk-clotting activity of the *O. tauricum* extract, a control test tube was prepared for each run without adding the RE to the milk.

## 3. Results and Discussion

Table 1 summarizes the physicochemical characteristics of the milks used in the experiment.

Cow’s milk presented a slightly lower pH value (6.67) than goat’s and ewe’s milk (pH 6.72–6.76). The different contents of nutrients reflected the species specificities for genuine milks. As the skimming had no or very little effect on the rennet coagulation time in cow’s, ewe’s, and goat’s milk [22], we used partially skimmed milks to reduce foaming and better catch the beginning of flocculation. In most of the experimental studies, the clotting properties of vegetable extracts were studied on reconstituted milk prepared from commercial bovine skim milk powder [8,23,24,25,26,27,28,29,30,31,32,33,34,35,36], while only a few authors used different substrates, namely whole and low-fat pasteurized milk [37,38,39,40], and thermized (55 °C for 15 s) milk [41]. To the authors’ knowledge, only Liburdi et al. [41] compared the clotting performance of vegetable extracts in milk of different origin (bovine, buffalo, goat, and ewe).

The flower heads of *O. tauricum* yielded 8.23 g of dry extract/100 g of fresh flowers. The total protein content measured on the RE was 3.61 µg/µL, while the total protein content of the liquid bovine rennet used as reference was 2.64 µg/µL. On the basis of the protein contents of milks and the volume of coagulants used, the ratios between milk protein and clotting enzymes were 97–161, 100–166, and 163–189 for cow’s, goat’s, and ewe’s milk, respectively. The dried plant extract contained 1.75 mg/100 mg of polyphenols. Some polyphenols that are specific to vegetable coagulants have been detected in curd and cheese, thus suggesting an innovative approach for the authentication of cheeses coagulated with plant extracts. Polyphenols could also confer healthy effects, as antioxidants, to the final products [42]. However, Barros et al. [43] underlined that the oxidized derivatives of phenolic compounds could inactivate the proteolytic enzymes, thus leading to a decrease in the plant extract activity. Undoubtedly, the technological properties of the vegetable CEs depend on the combination of specific and non-specific proteases. However, the enzymatic purity is usually of less importance than the costs and the ease of preparation and utilization of vegetable rennet. For these reasons, the characterization of the whole thistle extract, the study of its behavior in the milk of different species, and the comparison with the performance of commercial rennet are essential in the perspective of a real exploitation of *O. tauricum* aqueous extracts for cheesemaking.

The milk clotting activity was preliminary checked in crude extracts of different parts of the inflorescence (tubular flowers, receptacle), and of the stem and leaves. Even if two different kinds of extraction methods were tested (water, acetate buffer pH 5.0), no clotting activity after 120 min was detected in the receptacle and leaves extracts. Furthermore, no clotting activity was detected in the flower extracts upon heat treatment (100 °C for 5 min), clearly indicating the enzymatic nature of the milk coagulation.

Calcium ions play an essential role in the aggregation of casein micelles and in curd firmness. For this reason, the addition of CaCl_2_ to milk, especially to thermally processed ones, is the simplest way to reduce the clotting time and increase the curd firmness during cheesemaking. According to Kethireddipalli and Hill [44], a fortification with 0.1–0.2 g/L of CaCl_2_ (1–2 mM) is sufficient to obtain an optimal clotting of pasteurized milk, but up to 0.3–0.6 g/L (3–6 mM) are needed for milks that were subjected to more intense heat treatments. In order to evaluate the effect of CaCl_2_ on clotting properties, the RE of *O. tauricum* (300 µL) was tested in 5 mL of the different milks at their natural pH (Table 1) and the temperature 35 °C, with and without a 10 mM CaCl_2_ addition, a concentration level that was previously tested by several authors [6,7,8,23,26,29,30,32,38,45,46]. The addition of 10 mM CaCl_2_ reduced the clotting time of ewe’s, goat’s, and cow’s milk by 3-fold (24 vs. 8 min), 8.3-fold (200 vs. 24 min), and 13.6-fold (82 vs. 6 min), respectively. As the clotting times of non-fortified goat’s and cow’s milk largely exceeded the useful value for cheesemaking, all experiments were carried out with calcium fortification. The consistent improvement in the clotting performance of thistle extract in goat’s and cow’s milk could be ascribed to the severity of the thermal process that they underwent. The impaired rennet clotting properties (longer clotting time, weaker curd) of heat-treated milks have been mainly attributed to the interactions between denatured whey proteins and casein micelles, which interfere with the micelle aggregation. No significant differences were observed in the breakdown of κ-casein between the heated and raw milk. A decrease in the soluble calcium concentration when milk was heated at a temperature higher than 90 °C was also found to negatively affect casein micelle aggregation [44]. However, mild heat treatments (pasteurization) had no significant effect on rennet coagulation time and curd firmness [47]. Calvo [22] confirmed that pasteurization (70 °C for 30 min) had no or very little effect on ewe’s and goat’s milk, but he also reported that the same treatment doubled the clotting time of cow’s milk. Experimental data about the effect of homogenization on milk coagulation were inconsistent. Although homogenization worsened the rheological properties of curd [48], a shorter clotting time was measured for homogenized milk than for skim milk [47,49].

Most of the experimental studies on the effect of the independent variables (pH, temperature, CE volume, calcium ion concentration, sodium chloride concentration) on clotting activity used a univariate approach [24,26,28,29,30,31,32,33,35,36,41], disregarding how response (MCA) could be affected by the interactions among variables. Guiama et al. [34] used a factorial experiment design, but with a different goal, i.e., optimizing the extraction parameters (fruit percentage, NaCl concentration, extraction temperature) to maximize the coagulant strength of *Solanum aethiopicum* fruit extract.

The generated reports of the RSM are summarized in Table 2, Table 3 and Table 4.

The quality of the fit to the polynomial models was checked by the regression coefficient R^2^, which measures the amount of total variability explained by the model, and the adjusted R^2^, which shows the percentage of variation explained by only the independent variables (T, pH, volume of coagulant) that significantly affect the dependent variable (MCA) (Table 4). The results showed that the second-order model (Equation (2)) was significant for all milks, clotted by both the plant extract and animal rennet. Fisher’s F-test and *p*-value showed the significance of each coefficient (Table 2 and Table 3). It was observed that the volume of coagulant and, in a more general way, the milk/coagulant ratio, did not influence in a significant way the MCA of both the vegetable and animal rennet in all milks. The clotting properties of *O. tauricum* extracts in ewe’s milk were strongly affected by pH and the interaction T × pH, while the latter factor alone characterized the behavior of calf rennet in ewe’s milk. In goat’s milk, negative coefficients for pH and T × pH demonstrated linear and interactive effects to increase MCA, as well as the positive coefficients for pH × pH revealed a quadratic effect to increase the MCA of both the thistle extract and calf rennet. Two effects (T × T and T × pH) had *p*-values less than 0.05 in cow’s milk added by plant extract, indicating they had a significant influence on the MCA, while only the quadratic pH affected the MCA of animal rennet in cow’s milk.

**Figure 2:**
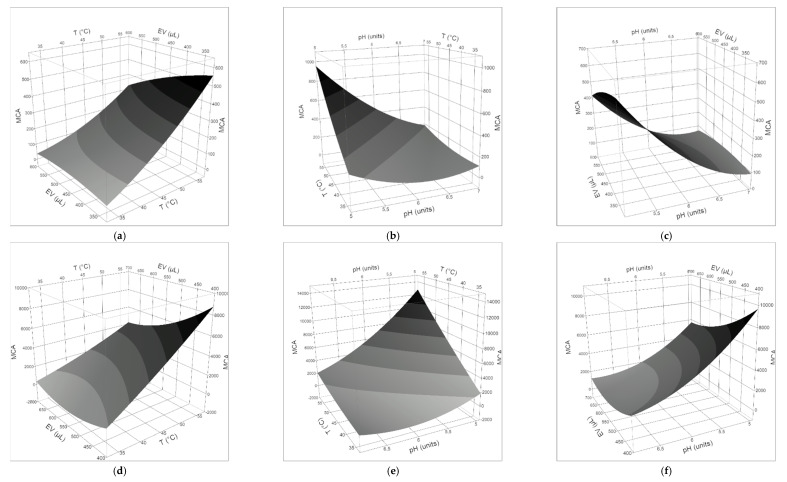
Response surface plots for the effects of (**a**) coagulant volume and temperature; (**b**) pH and T; (**c**) pH and coagulant volume, on the clotting activity of *O. tauricum* extract in ewe’s milk; (**d**) coagulant volume and temperature; (**e**) pH and T; (**f**) pH and coagulant volume, on the clotting activity of commercial rennin in ewe’s milk.

**Figure 3:**
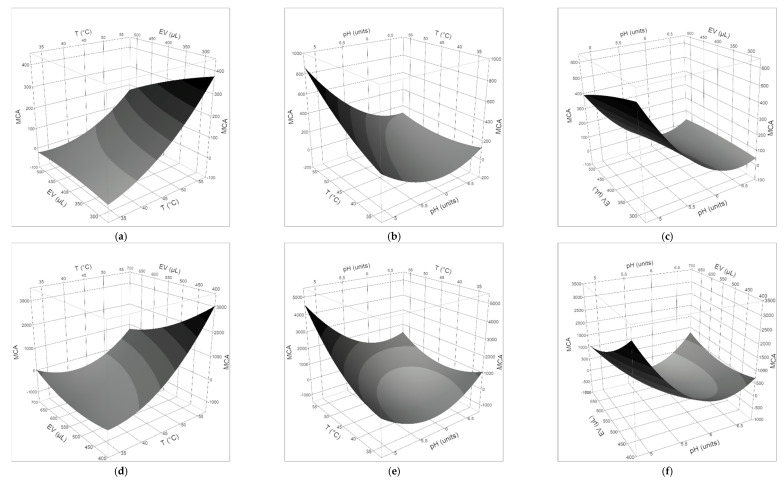
Response surface plots for the effects of (**a**) coagulant volume and temperature; (**b**) pH and T; (**c**) pH and coagulant volume, on the clotting activity of *O. tauricum* extract in goat’s milk; (**d**) coagulant volume and temperature; (**e**) pH and T; (**f**) pH and coagulant volume, on the clotting activity of commercial rennin in goat’s milk.

**Figure 4:**
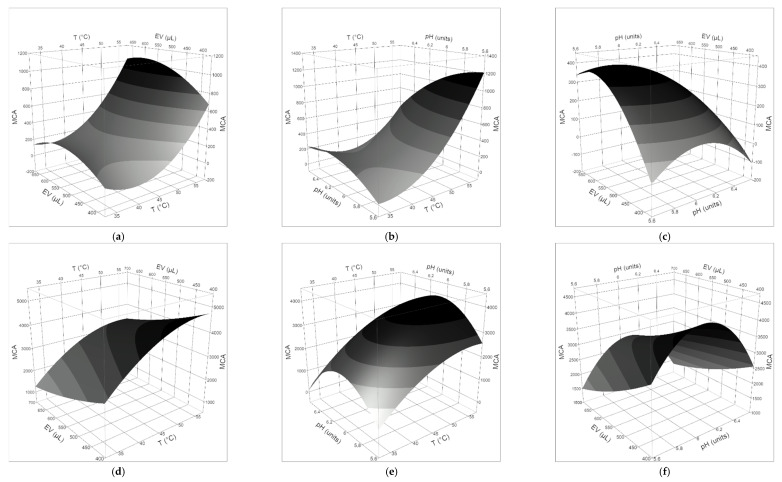
Response surface plots for the effects of (**a**) coagulant volume and temperature; (**b**) pH and T; (**c**) pH and coagulant volume, on the clotting activity of *O. tauricum* extract in cow’s milk; (**d**) coagulant volume and temperature; (**e**) pH and T; (**f**) pH and coagulant volume, on the clotting activity of commercial rennin in cow’s milk.

The three-dimensional plots of the effect of temperature, pH, and volume of coagulant on the MCA are given in Figure 2, Figure 3 and Figure 4. The findings highlighted that, within the explored range of the independent variables, a negative interaction between pH and temperature affected the general behavior of the MCA: the two variables had to move in opposite directions to cause a strong increase in the MCA. From a qualitative viewpoint, the clotting properties of ewe’s and goat’s milks appeared more affected by the kind of milk than the type of clotting agent, while the quadratic pH effect on the clotting of cow’s milk by calf rennet was reflected by the dome-shaped surface with a maximum in the pH range 5.8–6.2.

MCA describes the ability of the enzyme/extract to specifically hydrolyze the Phe_105_–Met_106_ bond of κ-casein, thus causing the destabilization of casein micelles, which in turn results in their aggregation. Therefore, the production of coagulants with a high specific MCA and the optimization of the clotting conditions are always the first goals to achieve. The desirability function was used to maximize the MCA and to estimate the predicted responses (Table 5). The input variables were kept within the ranges studied.

In all the milk/coagulant systems, the optimal temperature for clotting was the highest (55 °C) in the range explored (35–55 °C) and the optimal pH value (4.9–6.1) was the lowest in the actual range studied (5.0–6.9 for ewe’s milk, 5.7–6.5 for cow’s milk, 4.9–6.7 for goat’s milk). Interestingly, a general decrease in the MCA was observed by increasing the volume of coagulant, so that the optimal range for this parameter was 300–400 μL. The behavior of the thistle extract in cow’s milk was an exception: the higher the volume of the extract, the higher the clotting activity, so that the optimal value for this parameter was the highest used (500 μL). In the conditions that maximized the clotting activity, the performance of calf rennet was 5.0, 6.5, and 15.0 times better than thistle extract in cow’s, goat’s, and ewe’s milk, respectively. At the temperature of 35 °C and the natural pH of studied milks (Table 1), the estimated MCA of the *O. tauricum* extract was 72–87, 69–86, and 75–151, in goat’s, ewe’s, and cow’s milk, respectively, and in the range of the coagulant volume used. In comparison, the MCA of calf rennet was 5.4–4.9, 3.3–14.7, and 4.9–16.7 times higher than plant extract in goat’s, ewe’s, and cow’s milk, respectively.

As previously summarized, thermal and mechanical treatments have opposite effects on the time for milk casein to clot. Ewe’s milk should be the better substrate for specific proteases, as it was crude and it had the highest protein content (Table 1) and the more favorable casein/whey protein ratio [50]. By contrast, cow’s and goat’s milk underwent both a mechanical (homogenization) and a thermal (UHT) process. As a result, the range of the measured clotting times (30–360 s, 35–480 s, 47–1440 s, for cow’s, ewe’s, and goat’s milk, respectively) of the calculated MCAs (60–802, 66–994, 25–851 for the cow’s, ewe’s, and goat’s milk, respectively) and of the previously reported estimated MCA values at 35 °C were comparable for all the milk types used in the experiment.

Comparisons with the literature data suffer from the different definitions of “unit of enzymatic activity” and the different kind of substrate tested (milk species; reconstituted milk powder; crude, thermized or pasteurized milk), as also highlighted by Esteves et al. [51]. Therefore, the performance of *O. tauricum* extract was compared with that of commercial calf rennet in the same experimental conditions. Several authors applied to substrates made up of reconstituted skim milk powder the same definition of MCA that was adopted in the present study [8,28,31,33,34,35,36]. Particularly, Brutti et al. [8] reported a much lower clotting activity (1400 times lower than chymosin) for a partially purified extract of *O. acanthium* (30 °C, pH 6.5, 10 mM CaCl_2_). However, the same authors highlighted that “onopordosin” showed a more favorable clotting/proteolytic ratio than the aspartic protease of *C. cardunculus* and of proteases from other plants (*Asclepias fruticosa*, *Bromelia balansae*, *Bromelia hieronymi*, *Philibertia gilliesii*). A stronger clotting activity was observed by Anusha et al. [35] in latex (MCA 450) and stem, leaves, and flowers (MCA 29–319) of *Calotropis gigantea* (pH 5.5, 35 °C), and by Farias et al. [36] (MCA 239) in an extract of *Morinda citrifolia* L. (noni) fruit (pH 6.5, 37 °C). High values of MCA, exceeding those of chymosin (3.4 times), were found in quixaba (*Sideroxylon obtusifolium*) latex (10 μM CaCl_2_, 37 °C) [33]. The clotting performance of *O. tauricum* aqueous extract was consistent with the findings of Chazarra et al. [28] in crude extracts of dried flowers of artichoke (*Cynara scolymus* L.) (clotting times 100–500 s, 30–60 °C, pH 5.5–7.0), and of Guiama et al. [34] in extract produced from *Solanum aethiopicum* fruits (MCA 30–140). Mazorra et al. [38] used a different substrate (low-fat pasteurized milk, 0.02% CaCl_2_) for assessing the clotting properties of water extracts from kiwifruit (*Actinidia deliciosa*) and ginger (*Zingiber officinale*) and of melon (*Cucumis melo*) juice, and found that the MCA of chymosin was 67–120 times higher than vegetable extract activities. The clotting performance of *O. tauricum* extract was difficult to compare with that of the most studied species, *Cynara cardunculus*. In fact, several authors adopted a different definition of the unit of clotting activity (namely, amount of crude extract needed to coagulate 10 mL of reconstituted skim milk powder at 30 °C in 100 s) and reported values in the ranges 2–4.5 [7], 6–32 [23], 0.118–0.347 [45], and 0.092–0.512 [52]. Silva et al. reported specific MCAs (referred to 1 g of total protein content) [25] of 74.6 and 104, according to two concentration levels of ammonium sulfate added to crude extracts as precipitating agent, and the clotting times [27] of cardosins A and B (303–4384, 378–3615, and 230–4187 s, respectively) when they were added to reconstituted milk at the final concentration in the range 0.25–5.00 mg/mL.

Even if a balanced breakdown of caseins into small peptides and free amino acids is necessary for the proper development of the sensory properties of cheese during ripening, the excessive non-specific proteolytic activity causes low curd yields and sensory defects in cheese (bitter flavor, softness), thus limiting the use of most plant coagulants in cheese manufacturing, [1,8,52]. Therefore, the non-specific proteolytic activity of *O. tauricum* extract needs to be investigated and quantified. Inhibitors should also be tested to elucidate the nature of the catalytic type of the thistle proteases involved in the milk clotting.

## 4. Conclusions

For the first time, the clotting properties of an aqueous extract from *Onopordum tauricum* Willd. (Taurian thistle, bull cottonthistle) flowers were tested in milk of different origin (cow, goat, ewe) and compared with the clotting performance of commercial calf rennet. No clotting activity was detected in both aqueous and acidic (pH 5.5) crude extracts of other aerial anatomical parts (receptacle, leaves, stems). In addition, heat (100 °C for 5 min) wiped out the clotting properties of the flower extract, clearly indicating the enzymatic nature of the milk coagulation.

According to a second-order response surface model, the combination of operative parameters that maximize the clotting activity of *O. tauricum* extract was obtained at a temperature of 55 °C, pH of 4.9–5.7, and volume of coagulant equal to 300–500 μL. Under these optimal conditions, the predicted MCA was 798–1005, with the highest value measured in crude ewe’s milk. The clotting activity of Taurian thistle decreased as the temperature decreased (from 55 to 35 °C) and the pH increased (from 4.9 to 6.8). The estimated MCA of the *O. tauricum* extract at 35 °C and natural milk pH was consistent with the performance of the crude extracts of dried flowers of artichoke (*Cynara scolymus* L.) [27] and of *Solanum aethiopicum* fruits [33].

To better understand the technological behavior of *O. tauricum* extracts, comparative studies of caseinolytic and non-specific proteolytic activities must be carried out and completed with studies on rheological properties of milk gels and on sensory properties (texture, flavor, color) of ripened cheeses.

## Figures and Tables

**Table 1:** Physicochemical characteristics of the different milks used for the assessment of the clotting activity of aqueous extracts from *Onopordum tauricum* flowers.

Milk	pH	Dry Matter % w/w	Protein % w/w	Fat % w/w	Lactose % w/w	Ash % w/w
Bovine ^1^	6.67	12.2	3.5	1.6	5.8	1.3
Goat ^1^	6.72	10.8	3.6	1.6	4.8	0.8
Ewe ^2^	6.76	13.9	5.9	1.8	5.3	0.9

^1^ UHT. ^2^ raw.

**Table 2:** Estimated coefficients of the predicted second-order polynomial model for the milk clotting activity of *O. tauricum* extract.

Term	Ewe’s Milk			Goat’s Milk			Cow’s Milk		
	Estimates	F Ratio	*p*-Value	Estimates	F Ratio	*p*-Value	Estimates	F Ratio	*p*-Value
Intercept	2105.10			5553.6 *			−32,483.55		
Linear									
T	83.30	4.66	0.0563	57.42	3.10	0.1087	39.73	0.23	0.6493
EV	0.34	0.01	0.9107	−0.34	0.01	0.9129	14.48	5.14	0.0726
pH	−1145.98	4.75	0.0543	−2153.02	21.90	0.0009 *	9198.39	1.77	0.2408
Quadratic									
T × T	0.29	0.50	0.4962	0.43	1.58	0.2367	2.01	12.12	0.017 *
EV × EV	−0.00	0.16	0.6989	−0.00	0.05	0.8205	−0.01	4.68	0.0827
pH × pH	114.41	7.37	0.021 *	202.00	27.55	0.000 *		1.06	0.3504
Interactions									
T × EV	−0.03	3.07	0.1104	−0.03	2.82	0.1238	0.04	2.32	0.1885
T × pH	−13.05	39.00	<0.0001 *	−12.27	40.19	<0.0001 *	−34.89	12.32	0.017 *
EV × pH	0.30	2.75	0.1281	0.35	3.38	0.0957	−1.25	2.75	0.1581

* Level of significance *p* < 0.05. EV, volume of coagulant.

**Table 3:** Estimated coefficients of the predicted second-order polynomial model for the milk clotting activity of commercial calf rennet.

Term	Ewe’s Milk			Goat’s Milk			Cow’s Milk		
	Estimates	F Ratio	*p*-Value	Estimates	F Ratio	*p*-Value	Estimates	F Ratio	*p*-Value
Intercept	31,246.16			47,947.18 *			−211,167.38		
Linear									
T	1508.17	3.59	0.0873	193.76	0.20	0.6669	362.66	1.25	0.3145
EV	−59.52	1.31	0.2791	−22.67	0.56	0.4723	−19.89	0.65	0.4558
pH	−13,742.13	1.62	0.2316	−15,695.82	6.58	0.0282 *	69,645.77	6.49	0.0514
Quadratic									
T × T	1.71	0.04	0.8435	5.44	1.43	0.2594	−3.86	2.85	0.1524
EV × EV	0.04	0.97	0.3476	0.01	0.37	0.5579	0.01	0.25	0.6390
pH × pH	1395.31	2.60	0.1378	1411.55	7.59	0.0203 *	−5846.04	6.61	0.0500 *
Interactions									
T × EV	−0.55	2.78	0.1262	−0.35	3.53	0.0898	−0.01	0.01	0.9145
T × pH	−187.60	18.96	0.0014 *	−69.72	7.24	0.0227 *	12.30	0.10	0.7672
EV × pH	4.83	2.32	0.1588	3.26	3.01	0.1134	1.39	0.23	0.6505

* Level of significance *p* < 0.05. EV, volume of coagulant.

**Table 4:** Models for the milk clotting activity (MCA).

	MCA ^1^	R^2^	Adjusted R^2^	F Ratio	*p*-Value
Ewe’s milk					
RE	=114.41 (pH)^2^ − 13.05 (T) (pH)	0.9648	0.9330	30.43	<0.0001 *
CR	=−187.60 (T) (pH)	0.9315	0.8698	15.11	0.0001 *
Goat’s milk					
RE	=5553.69 − 2153.02 (pH) − 12.27 (T) + 202.00 (pH)^2^	0.9698	0.9427	35.72	<0.0001 *
CR	=−47,947.18 − 15,695.82 (pH) − 69.72 (T) (pH) + 1411.55 (pH)^2^	0.8622	0.7383	6.95	0.0028 *
Cow’s milk					
RE	=2.01 (T)^2^ − 34.89 (T) (pH)	0.9636	0.8980	14.70	0.0043 *
CR	=−5846.04 (pH)^2^	0.9701	0.9163	18.03	0.0027 *

^1^ Each model equation is presented using the significant experimental values (*p* < 0.05). RE, reconstituted extract of *O. tauricum* flowers. CR, commercial calf rennet. * Level of significance *p* < 0.05. EV, volume of coagulant.

**Table 5:** Optimal conditions for milk clotting.

	T (°C)	EV (μL)	pH (Units)	Predicted MCA ^1^	Desirability	Measured MCA
Ewe’s milk + RE	55	300	5.0	1005	0.9852	989
Ewe’s milk + CR	55	400	4.9	15,015	0.9829	14,634
Goat’s milk + RE	55	300	4.9	798	0.8711	821
Goat’s milk + CR	55	400	4.9	5155	0.8645	5254
Cow’s milk + RE	55	500	5.7	940	0.9946	892
Cow’s milk + CR	55	400	6.1	4775	0.9965	4651

^1^ Maximize desirability. EV, volume of coagulant. RE, reconstituted extract of *O. tauricum* flowers. CR, commercial liquid calf rennet.

## Supplementary Materials

The following are available online at https://www.mdpi.com/2304-8158/9/6/692/s1, Table S1: Experimental design matrix (D-optimal criterion) and observed responses (clotting time) in cow’s, goat’s, and ewe’s milk.
