# Supplementary material for: Clotting Properties of Onopordum tauricum (Willd.) Aqueous Extract in Milk of Different Species
Source: Foods. 2020 May 27;9(6):692. doi: 10.3390/foods9060692 (PMC7353650; doi:10.3390/foods9060692)
Supplement: Supplementary file 1 [file foods-09-00692-s001.pdf]

Table S1. Experimental design matrix (D-optimal criterion) and observed responses (clotting time) in cow's, goat's, and ewe's milk.

		Cow's milk[2]			Goat's milk			Ewe's milk		
**Run**	**T**	**EV**	**pH**	**CT[1]**	**EV**	**pH**	**CT[1]**	**EV**	**pH**	**CT[1]**
***Onopordum tauricum* extract**										
1	53	417	6.45	100	320	6.5	320	368	6.9	195
2	35				500	4.9	130	575	5.05	90
3	35	430	6.5	360	330	6.7	1440	379	6.9	480
4	38				350	4.9	100	402	5.05	80
5	55	630	6.5	35	480	6.7	500	554	6.9	180
6	50				470	4.9	53	540	5.05	40
7	47	417	5.7	147	320	5.7	390	368	5.95	115
8	52	650	6.5	90	500	6.7	570	575	6.9	200
9	55	459	6.06	35	350	6.1	190	402	6.87	100
10	42	643	5.7	80	490	5.7	455	563	5.95	120
11	47	499	6.45	110	380	6.5	670	437	6.9	238
12	35	630	6.45	320	480	6.5	920	554	6.9	297
13	42	565	6.5	200	430	6.7	870	494	6.9	255
14	55				300	4.9	47	345	5.05	35
15	55	390	6.5	110	300	6.7	377	345	6.9	250
16	38	390	6.45	320	300	6.5	900	345	6.9	320
17	36	565	5.7	140	430	5.7	390	494	5.95	213
18	37	650	6.5	250	500	6.7	930	575	6.9	360
19	53	499	5.7	30	380	5.7	180	437	5.95	85
20	35				300	4.9	160	345	5.05	130
**Commercial calf rennet (liquid form)**										
1	53	438	6.45	8	438	6.5	25	438	6.9	11
2	35	680			680	4.9	35	680	5.05	5
3	35	451	6.5	19	451	6.7	80	451	6.9	37
4	38	480			480	4.9	25	480	5.05	10
5	55	655	6.5	7	655	6.7	17	655	6.9	12
6	50	640			640	4.9	15	640	5.05	3
7	47	438	5.7	8	438	5.7	600	438	5.95	5
8	52	680	6.5	8	680	6.7	30	680	6.9	14
9	55	478	6.06	6	478	6.1	18	478	6.87	8
10	42	670	5.7	10	670	5.7	900	670	5.95	26
11	47	520	6.45	7	520	6.5	45	520	6.9	10
12	35	656	6.45	18	656	6.5	65	656	6.9	28
13	42	588	6.5	13	588	6.7	50	588	6.9	20
14	55	410			410	4.9	5	410	5.05	2
15	55	410	6.5	8	410	6.7	20	410	6.9	13
16	38	410	6.45	11	410	6.5	85	410	6.9	18
17	36	588	5.7	14	588	5.7	1800	588	5.95	150
18	37	680	6.5	16	680	6.7	55	680	6.9	31
19	53	520	5.7	7	520	5.7	1920	520	5.95	5
20	35				410	4.9	40	410	5.05	7

[1] Average values of two replicates of each run. [2] Runs n. 2, 4, 6, 14, and 20 were not investigated in cow milk due to casein precipitation in acetate buffer at pH 4.5.

T = temperature (°C), EV = volume of coagulant (μL), CT = clotting time (s).
